# Arterial Stiffness in Balkan Endemic Nephropathy, an Environmental Form of Aristolochic Acid Nephropathy

**DOI:** 10.3389/fcvm.2018.00166

**Published:** 2018-11-15

**Authors:** Vedran Premužić, Vanja Ivković, Ninoslav Leko, Želimir Stipančić, Sandra Karanović, Ana Jelaković, Ivana Vuković Brinar, Živka Dika, Bojan Jelaković

**Affiliations:** ^1^Department of Nephrology, Hypertension, Dialysis and Transplantation, School of Medicine, University Hospital Centre Zagreb, University of Zagreb, Zagreb, Croatia; ^2^General Hospital “Dr. Josip Benčević” Slavonski Brod, Croatia; ^3^Department of Dialysis Odžak, County Hospital Orašje, Odžak, Bosnia and Herzegovina

**Keywords:** Balkan endemic nephropathy, aristolochic acid nephropathy, arterial stiffness, chronic hemodialysis, chronic kidney disease, pulse wave velocity

## Abstract

Balkan endemic nephropathy (BEN), an environmental form of aristolochic acid nephropathy is characterized with later onset and milder forms of hypertension (HT). Thus, we hypothesized that arterial stiffness progresses slower in BEN patients resulting in lower CV mortality. A total of 186 hemodialysed (HD) patients (90 BEN, 96 non-BEN; 67.3 + 13.0 years) were enrolled and followed-up for 25 months. Brachial blood pressure (BP) and pulse wave velocity (PWV) were determined before mid-week dialysis. BEN patients were older (72.1 ± 37.1 vs. 62.8 ± 15.1; *p* < 0.001), had shorter duration of HT prior commencement of HD than non-BEN patients (36 vs. 84 months; *p* < 0.001). There were no differences in BP, but BEN patients were treated with less antihypertensive drugs (*p* < 0.01). BEN patients had lower PWV values at baseline and at the end of follow-up period despite being chronologically older (*p* < 0.001). Baseline PWV > 10 m/s was associated with higher risk for CV mortality (aHR 1.8 [1.4, 2.4]). In multivariate analyses BEN was predictor of lower PWV. During the follow-up period significantly less CV deaths were observed in BEN vs. on-BEN patients (12 vs. 31; *p* = 0.001). CV mortality adjusted for other risk factors was significantly lower in BEN group (aHR 0.2 [0.1, 0.5]). Overall BEN patients had longer mean survival time on HD (22.3 vs. 18.2 months; *p* < 0.001). Observed slower vascular aging (i.e., lower PWV) in BEN patients compared to other ESRD patients is related to the later onset of HT and milder stages of HT during predialytic clinical course and better control of BP and phosphate during HD.

## Introduction

Cardiovascular disease (CV) is the most important cause of morbidity and mortality in chronic kidney disease (CKD) (1–6). It is well-established that the most characteristic aspect of arterial disease in CKD/ESRD is premature vascular aging. Mean arterial pressure and age are most important risk factors for increased arterial stiffness. Blacher et al. found increased pulse wave velocity (PWV), to be a predictor of all-cause mortality and CV mortality in patients with ESRD (7). This was confirmed by other authors analyzing data on patients in different CKD stages (3–9). Higher values of PWV were found already in the early stages of CKD and it was reported that arterial stiffness is also an independent risk factor for progression of CKD starting from CKD stage 3 (10–12). On the other hand, it was shown that improving PWV is associated with better survival in CKD patients independently of blood pressure (BP) level (3, 7, 8). In patients with autosomal dominant polycystic kidney disease arterial stiffness is evident before the onset of hypertension and decline of eGFR, so this form of CKD was proposed to be a case of exaggerated early vascular aging (13). It seems that different forms of CKD differ in arterial stiffness and progression of vascular aging. Balkan endemic nephropathy (BEN) is chronic tubulointerstitial nephropathy frequently associated with upper urothelial cancers. Recently, it was proven that BEN is an environmental form of worldwide present aristolochic acid nephropathy (14–17). Later onset of hypertension during the predialytic clinical course and mild stages of hypertension were reported to be important features of BEN (15, 17–20). The most plausible explanation is salt wasting. As mild and labile hypertension was occasionally observed in BEN and became more frequent only in advanced stages of CKD, the aim of our study was to determine possible differences in arterial stiffness between BEN and non-BEN patients undergoing chronic dialysis. Furthermore, we proposed that patients with BEN have lower CV morbidity and mortality than non-BEN ESRD patients. To test this hypothesis we analyzed PWV and clinical course of BEN and non-BEN patients undergoing chronic hemodialysis.

## Materials and methods

This was a prospective, longitudinal follow-up study of 25 months duration. In total, 186 patients were recruited from three dialytic units and two countries (General Hospital “Dr. Josip Benčević” Slavonski Brod, Croatia; General Hospital Odžak, Bosnia and Herzegovina; and University Hospital Centre Zagreb, Croatia). The participation in the study was approved by the local Ethical Committees. Inclusion criteria were: undergoing chronic hemodialysis for at least 3 months and signed informed consent. Exclusion criteria were: atrial fibrillation or other chronic arrhythmias, recent stroke (<6 months), transitory ischemic attack (TIA) or myocardial infarction (<6 months), congestive heart failure (NYHA III-IV), significant hemodynamic instability during the dialysis, presence of old artery-venous anastomoses at contralateral arm of the currently used one. Diagnosis of BEN and classification of subjects were done as described previously in the consensus document (16). At baseline, there were 90 BEN patients (37 men, 53 women) and 96 non-BEN patients (48 men, 48 women). In the non-BEN group the most frequent causes of ESRD were diabetes (26%) and hypertension (21.8%) followed by chronic glomerulonephritis (16.6%) and autosomal dominant polycystic kidney disease (8.3%). Diabetes was more frequently diagnosed in non-BEN than in BEN group (21 vs. 5; *p* = 0.001). Although at baseline substantially more patients in non-BEN group had hypertension (90 vs.79), coronary heart disease (32 vs. 22) and left ventricular hypertrophy (67 vs. 58) differences did not reach statistically significant level (*p* > 0.05). BEN patients had significantly shorter duration of hypertension prior commencement of hemodialysis than non-BEN patients [36 (12–84) vs. 84 (60–120) months; *p* < 0.001];. During the study period they were treated with significantly less antihypertensive drugs (1.22 ± 0.93 vs. 2.27 ± 1.32; *p* < 0.001), and were much more frequently controlled with monotherapy than non-BEN patients (43 vs. 21%; *p* = 0.0001). Non-BEN patients were treated more frequently with beta blockers, alpha blockers, loop diuretics, moxonidine, and minoxidile than EN patients (*p* < 0.001). There were no differences in frequency of usage of ACE inhibitors, ARBs, and calcium channel blockers (*p* > 0.05). Statins were used more frequently in non-EN group (27 vs. 13%; *p* = 0.02). Later in conducted analyses we failed to find signicicant impact of used antihypertensive class on the main findings i.e., on arterial stiffness possibly because most of non-BEN patients were treated with various drugs combination. All patients were on erythropoietin therapy and folic acid (B9), 82% were treated with calcitriol while 78% of patients were on phosphate-binders therapy (CaCO_3_ and sevelamer). Patients were dialyzed three times a week with standard bicarbonate hemodialysis solutions and synthetic dialysers, with blood flow rates of 300–350 ml/min and dialysate flow rates of 500–800 ml/min, aiming at a dialysis dose Kt/V > 1.2. There were no differences in type of vascular access between BEN and non-BEN group.

At the end of follow up period there were 52 BEN patients (20 men, 32 women) and 45 non-BEN patients (22 men, 23 women) non-BEN patients. Death was recorded in 28 and 37 BEN and non-BEN patients, respectively (31 vs. 39%; *p* = 0.29). During the study period 24 patients undergone kidney transplantation with no difference in proportion of patients between BEN vs. non-BEN groups (11.1 vs. 14.5%, *p* = 0.45). Transplanted patients were excluded from the cohort and analyses at the end of follow-up.

Data on medical history and medication were collected from hospital records. Total serum cholesterol, triglycerides, glucose, iron, uric acid, potassium, calcium, phosphate, red blood cell count, hematocrite, hemoglobin were analyzed from fasting blood samples. All patients were studied on the mid-week dialysis day after a 2-days interdialytic interval. Body mass index (BMI) was calculated as dry weight divided by the square of body height. The mean value of three measurements of the post-dialytic body weight was defined as dry weight. Brachial BP was measured before dialysis using Omron M6 device (Kyoto, Japan) in sitting position and was determined as mean of three measurements at the non-fistula arm. PWV and aortic augmentation index (Aix), were assessed by Tensiomed Arteriograph (Medexpert Ltd., Budapest, Hungary), a computerized device using an oscillometric method which simultaneously measure brachial BP, PWV and Aix. PWV and Aix were determined before dialysis as a mean of three measurements at the non-fistula arm. All measurements which were taken by the same physician (V.P.) in a quiet room after resting at least 15 min before the measurements. PWV value >10 m/s was used as a cut-off value as recommended by the European Society of Hypertension/European Society of Cardiology guidelines (21). Pulse pressure amplification was determined as an absolute difference between brachial and central pulse pressure while pulse pressure amplification ratio was determined as a ratio between brachial and central pulse pressure.

Statistical analysis was performed using SPSS version 23.0 (IBM Corp., USA). Normality of data distribution was tested using Kolmogorov-Smirnov test. Preliminary analyses were performed to ensure no violation of the assumptions of normality, linearity and homoscedasticity. Categorical data were expressed as numbers and frequencies. Correlations were obtained using Pearson's test for normally distributed variables and Spearman rank correlation for non-normally distributed variables. Normally distributed variables were presented as means ± standard deviations and Student's *t*-test for independent samples was used for comparisons between two groups. Non-normally distributed data were presented as median and interquartile range and Mann-Whitney U-test was used in comparison between two groups. Baseline-to-follow-up comparisons were done using Student's *t*-test for paired samples and Wilcoxon test. Categorical variables were compared using χ^2^-test. Survival analysis was done with Kaplan-Meier curves which were tested with log-rank test while hazard ratios were estimated with Cox proportional hazards regression. Multiple linear regression was used to explore the influence of different variables on PWV, while logistic regression was used for categorical dependent variables. We constructed three linear regression models to assess independent associations of multiple independent variables with PWV. In Model 1 we included variables known to be associated with arterial stiffness: age, gender, mean arterial pressure (MAP), hematocrite, ultrafiltration, vitamin D dose, phosphate binders dose, CaxP, vascular access type, heart rate, and BEN status (yes/no). Model 2 included, in addition to all variables in Model 1 those further related to the dialysis: dialysis vintage, Kt/V, sevelamer (yes/no), and erythropoietin dose per kilogram of weight. Model 3 was additionally adjusted for established CV risk factors: BMI, smoking (yes/no), hypertension (yes/no), and DM (yes/no). A *p* < 0.05 (two-sided tests) was considered significant.

## Results

Demographic and clinical characteristics of enrolled patients at baseline and the end of follow-up are shown in Table 1. There were no differences in gender and smoking status between BEN and non-BEN groups. Importantly, BEN patients were older at baseline and the end of follow-up (both *p* < 0.01). They were also older when started with dialysis (*p* < 0.001). No significant differences were observed in dialysis vintage. Obesity and visceral obesity were more frequently present in non-BEN group. There were no differences in dialytic parameters between two groups beside ultrafiltration which was significantly higher in non-BEN group (*p* < 0.001). We failed to detect differences in daily phosphate binder, calcium load, vitamin D weekly dose, and usage of sevelamer between two groups. As shown in Table 1 at baseline and at the end of follow-up BEN patients had lower values of serum phosphate, calcium, product of calcium and phosphate, and iPTH.

**Table 1:** Demographic, clinical, and laboratory data of enrolled patient at baseline and at the end of follow-up.

	**Baseline**			**Follow-up**		
	**BEN**	**Non-BEN**	***p***	**BEN**	**Non-BEN**	***p***
Number of patients	90	96		52	45
Men/women	37/53	48/48	0.285	20/32	22/23	0.407
Age (years)	73 (68–78)	66 (54–75)	<0.001	75 (71–78)	68 (56–75)	0.002
Age on start of dialysis (years)	67 (62–72)	57.5 (42.3–68.8)	<0.001	69 (64–74)	60 (45.2–70.2)	<0.001
Body height (cm)	165 + 8	168 + 10	0.036	165 + 7	167 + 8	0.18
Body weight (kg)	63 + 12	70 + 14	<0.001	62 + 10	68 + 13	0.014
Body mass index (kg/m^2^)	23.1 + 3.6	24.9 + 4.4	0.003	22.9 (19.2–29.3)	24.8 (20.1–31.2)	0.02
Waist circumference (cm)	78 (70–84)	82 (78–91)	<0.001	77 (69–85)	82 (74–90)	0.009
Smokers (%)	13.3	13.5	0.96	11.2	11.5	0.92
Dialysis vintage (months)	68 + 49	82 + 63	0.09	99 + 52	102 + 61	0.83
Vascular access (A-V fistula/CVC)	73/17	72/24	0.31	40/12	38/7	0.28
Duration of dialysis (h)	4.3 (4.0–4.5)	4.0 (3.5–4.5)	0.61	4.2 (3.7–4.7)	3.8 (3.3–4.3)	0.21
Ultrafiltration (ml)	3,500 (2,500–4,000)	4,000 (3,500–4,000)	<0.001	3,220 (2,700–3,700)	3,620 (3,000–4,000)	<0.001
Residual diuresis (ml)	330 (240–420)	380 (310–470)	0.43	335 (285–385)	302 (250–350)	0.69
Kt/V	1.3 + 0.4	1.2 + 0.3	0.68	1.3 + 0.4	1.2 + 0.3	0.58
Weekly/weight erythropoietin load (IU/kg)	134 + 54	118 + 47	0.07	124 + 24	123 + 21	0.74
Daily phosphate binder calcium load (g/day)	3.2 + 1.6	2.8 + 1.6	0.28	2.1 + 2.01	2.1 + 2.1	0.88
Sevelamer (%)	16.6	27.1	0.08	17.9	29.1	0.07
Weekly vitamin D load μg/week	0.8 + 0.5	0.8 + 0.7	0.99	0.6 + 0.5	0.8 + 0.4	0.19
RBC (10^12^/l)	3.6 (3.2–3.9)	3.3 (3.1–3.6)	0.001	3.3 (3.1–3.7)	3.5 (3.3–3.8)	0.19
Hemoglobin (g/l)	115 (108–127)	108 (100–115)	<0.001	113 (102–124)	111 (102–124)	0.68
Serum calcium (mmol/l)	2.2 ± 0.1	2.3 ± 0.2	0.23	1.2 (1.1–2.2)	2.27 (2.2–2.3)	<0.001
Serum phosphate (mmol/l)	1.3 ± 0.4	1.6 ± 0.4	<0.001	1.2 ± 0.2	1.4 ± 0.4	0.014
Ca × P	3.1 ± 1.1	3.7 ± 1.1	<0.001	2.1 ± 1.1	3.4 ± 1.2	<0.001
iPTH (pmol/l)	10.4 (4.5–19.6)	20.2 (12.0–50.0)	<0.001	18.4 (8.0–28.8)	32.7 (9.6–79.0)	0.013
Serum glucose (mmol/l)	5.3 (4.9–6.3)	5.5 (4.6–6.8)	0.88	5.7 (5.2–6.4)	5.7 (5.2–5.6)	0.55
Total serum cholesterol (mmol/l)	4.6 (4.1–5.1)	4.9 (3.9–5.2)	0.90	4.6 ± 1.1	4.1 ± 1.1	0.03
Serum triglycerides (mmol/l)	1.8 (1.3–2.3)	2.0 (1.8–2.3)	0.035	1.26 (0.99–1.81)	1.17 (1.02–2.12)	0.96
Serum uric acid (μmol/l)	322 (288–352)	317 (282–355)	0.61	346 ± 64	325 ± 59	0.09

*BEN, Balkan endemic nephropathy; A-V, fistula–arterio-venous fistula; CVC, central venous catheter; iPTH, intact parathormone; results are shown as mean ± SD or median (interquartile range)*.

There were no differences in brachial BP and pulse pressure values between BEN and non-BEN patients at baseline and at the end of follow-up (Table 2). PWV was significantly slower in BEN patients at baseline and at the end of follow-up (both *p* < 0.01). When adjusted for the same confounders included in Model 3 of linear regression PWV was still slower in BEN vs. non-BEN patients (*p* < 0.01). On univariate analysis PWV was correlated with AIx (*r* = 0.236; *p* = 0.001), dialysis vintage (*r* = 0.143; *p* = 0.05), duration of dialysis (*r* = −0.304; *p* < 0.001), systolic BP (*r* = 0.209, *p* = 0.004), diastolic BP (*r* = 0.188, *p* = 0.01), pulse pressure (*r* = 0.158, *p* = 0.03), MAP (0.212, *p* = 0.004), central systolic BP (*r* = 0.231, *p* = 0.001), central pulse pressure (*r* = 0.244, *p* = 0.001), and age in non-BEN patients (*r* = 0.31; *p* = 0.04). In all three linear regression models BEN was the strongest predictor of lower PWV (β = −0.525, β = −0.508, and β = −0.453, all *p* < 0.005). Additionally, in Model 1 (adjusted *R*^2^ = 0.212) MAP (β = 0.307, *p* = 0.005) showed positive association with PWV. In Model 2 (adjusted R^2^ = 0.288) MAP (β = 0.239, *p* = 0.05), age (β = 0.333, *p* = 0.04), hematocrite (β = 0.294, *p* = 0.03), and weekly/weight erythropoietin load (β = 0.293, *p* = 0.01) were positively associated with PWV. Model 3 (adjusted *R*^2^ = 0.306) showed positive significant associations of PWV with MAP (β = 0.267, *p* = 0.04), age (β = 0.317, *p* = 0.01), and weekly/weight erythropoietin load (β = 0.246, *p* = 0.05). On logistic regression, non-BEN patients had adjusted OR (adjusted as in Model 3 of linear regression) for having PWV > 10 m/s of 6.63 [2.19, 20.09] compared to BEN patients.

**Table 2:** Hemodynamic characteristics of BEN and non-BEN patients at baseline and follow-up.

	**Baseline**			**Follow-up**		
	**BEN**	**Non-BEN**	***p***	**BEN**	**Non-BEN**	***p***
Systolic BP (mmHg)	160 ± 31	155 ± 31	0.308	153 (139–174)	159 (141–172)	0.51
Diastolic BP (mmHg)	85 (77–96)	84 (76–96)	0.60	84 ± 17	84 ± 14	0.99
Mean BP (mmHg)	110 (97–122)	108 (95–122)	0.40	110 ± 21	111 ± 19	0.73
Heart rate (beats/min)	76 ± 13	72 ± 12	0.017	74 ± 13	71 ± 12	0.37
PP (mmHg)	75 ± 20	71 ± 22	0.36	69 (59–83)	75 (61–91)	0.44
Central PP (mmHg)	74 ± 26	77 ± 31	0.47	68.3 (53.7–80.6)	72.4 (57.2–89.5)	0.61
PP amplification	−4.75 (−8.56–5.56)	−7.2 (−9.45–1.60)	0.06	−4.58 (−8.26–5.31)	−7.32 (−9.12–1.8)	0.12
PP amplification ratio	0.92 (0.90–1.08)	0.91 (0.88–1.03)	0.02	0.98 (0.91–1.21)	0.89 (0.87–1.32)	0.01
Central systolic BP (mmHg)	161.4 ± 35.6	157.4 ± 35.0	0.43	158.3 ± 33.2	163.5 ± 34.1	0.45
AIx (%)	38.8 ± 16.5	39.0 ± 15.3	0.93	40.7 ± 15.3	43.4 ± 14.7	0.38
PWV (m/s)	9.2 ± 1.6	10.5 ± 1.9	< 0.001	9.3 ± 1.3	10.5 ± 1.9	0.001
adjusted PWV*	9.2 ± 0.2	10.7 ± 0.2	0.004	9.3 ± 0.6	10.7 ± 0.4	0.001

Results are shown as mean ± SD or median (interquartile range);

* *ANCOVA adjusted as in Model 3, mean ± standard error (SE) BEN, Balkan endemic nephropathy; BP, blood pressure; Aix, augmentation index; PWV, pulse wave velocity; PP, pulse pressure*.

**Figure 1:**
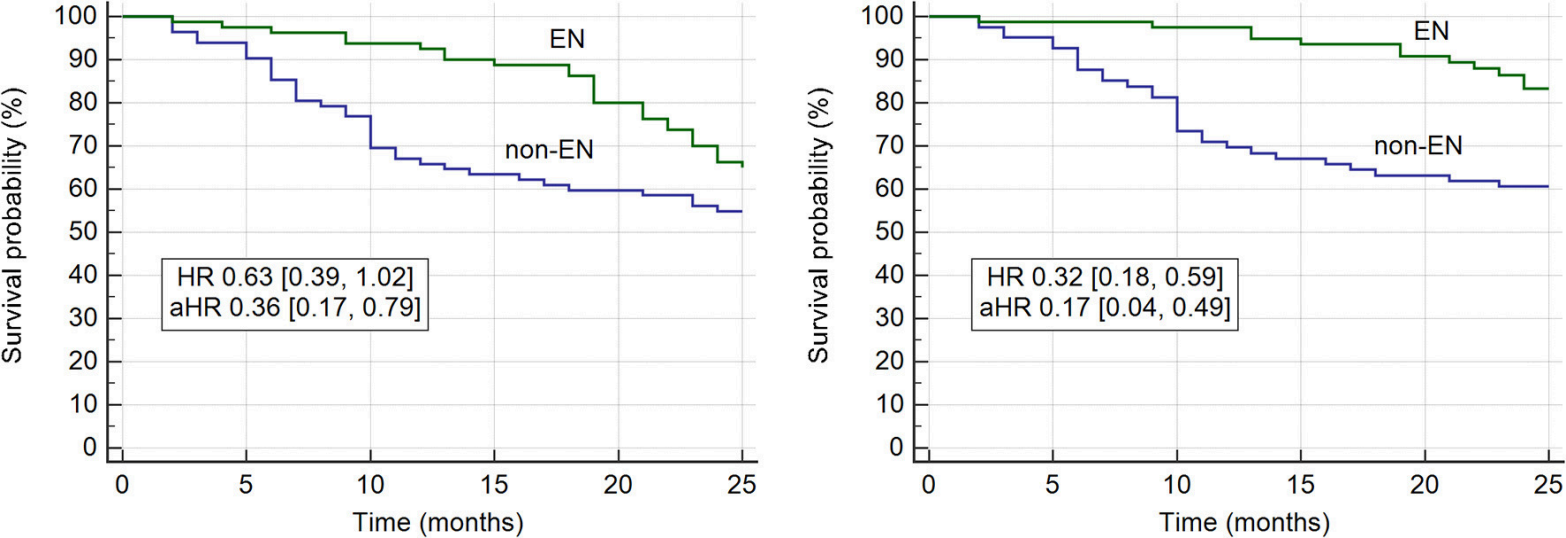
All-cause **(left)** and cardiovascular **(right)** mortality in BEN and non-BEN patients at the end of follow-up. BEN, Balkan endemic nephropathy.

**Figure 2:**
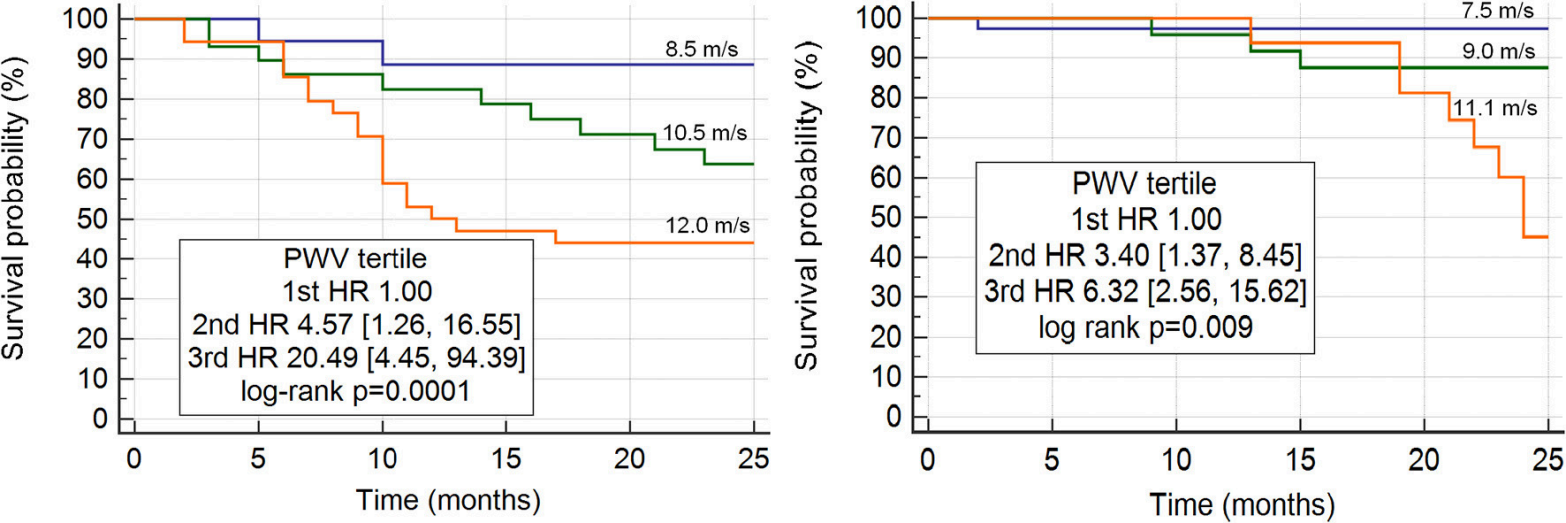
Increased aortic stiffness (PWV) is a strong independent predictor of cardiovascular mortality in non-BEN **(left)** and in BEN **(right)** patients undergoing hemodialysis. BEN, Balkan endemic nephropathy.

As shown in Table 3, in all decades lower average PWV values were found in BEN patients compared to non-BEN group. This is in line with the result on lower percentage of BEN patients with PWV values above the referral values compared to non-BEN patients what was found in all decades. In the BEN group only one patient was younger than 50 years (decade 40–49 years). Mean survival time on dialysis was longer in BEN group than in non-BEN group [22.3 (95% CI 21.2, 23.5) vs. 18.2 (95% CI 16.4, 20.0) months, *p* < 0.001]. Non-BEN patients died more frequently from CV events than BEN patients [31 (83.7%) vs. 12 (42.8%); *p* = 0.001]. Stroke, myocardial infarction and heart failure were causes of deaths in 21.6, 24.3, and 38.8% in non-BEN patients, respectively; and in 14.2, 10.7, and 17.8% BEN patients, respectively. There was a marginally significant difference in all-cause mortality between BEN and non-BEN patients (35.0 vs. 45.1%, HR 0.63 [0.39, 1.02], log-rank *p* = 0.057) which became significant after adjustment for multiple risk factors and probable confounders (listed in Model 3 of linear regression) (aHR 0.36 [0.17, 0.79]) (Figure 1). CV mortality was significantly lower in BEN group (15.0 vs. 37.8%, HR 0.32 [0.18, 0.59], log-rank *p* = 0.0004; aHR 0.17 [0.06, 0.49]) (Figure 1). Baseline PWV > 10 m/s was associated with higher adjusted hazard for both all-cause (aHR 2.14 [1.15, 3.97]) and CV mortality (aHR 1.88 [1.42, 2.49]) (Figure 2).

**Table 3:** Average values of PWV and proportion of BEN and non-BEN patients with PWV values above the cut off for Arteriograph in groups diveded into decades.

**Decade (years)**	**BEN (m/s)**	**Non-BEN (m/s)**	**BEN**		**Non-BEN**		**χ^2^ (*p*)**
			**N**	**%**	**N**	**%**
20–30	–	9.0 (6.9–11.6)	0/0	–	3/5	60	–
30–40	–	9.7 (6.9–12.2)	0/0	–	3/6	50	–
40–50	7.6	10.6 (8.8–13.0)	0/1	0	5/7	71.4	–
50–60	9.7 (8.6–11.5)	10.6 (7.5–13.0)	1/3	33.3	5/11	45.4	0.134 (0.70)
60–70	9.3 (6.7–13.2)	11.2 (7.8–15.7)	10/26	38.4	19/28	67.8	1.435 (0.23)
>70	9.1 (5.9–12.6)	10.6 (7.2–15.1)	18/60	30.0	24/37	64.8	4.296 (0.03)
Total			29/90	32.2	59/96	61.5	5.791 (0.01)

*BEN, Balkan endemic nephropathy*.

## Discussion

The most important result obtained in this study is finding that. BEN patients undergoing chronic hemodialysis had significantly lower PWV values at baseline and at the end of follow-up period than non-BEN patients despite being significantly older than non-BEN patients. The most plausible explanation is later onset and milder forms of hypertension during the predialytic clinical course of BEN, which was also observed by other authors (14, 19, 20). Lower PWV in BEN patients could be a biomarker of slower vascular aging and consecutive slower progression of CKD and lower CV risk in this group of ESRD patients. BEN patients were significantly older than non-BEN patients when started with dialysis what is in line with reports from other BEN dialytic units (22, 23). Recently in surveys conducted in Croatian endemic area we found that in predialytic period CV mortality was significantly lower in BEN than in non-BEN patients (Jelaković and Premužić, 2018, unpublished data). In this study hypertension was better controlled with less antihypertensive drugs in BEN patients compared to non-BEN patients. Furthermore, phosphate, calcium, and iPTH values were better controlled in BEN patients compared to other ESRD patients what contributed to less vascular calcification and slower progression of arterial stiffness during the follow-up period. In both groups, PWV was strong independent predictor of CV mortality, particularly deaths from stroke and heart failure (Figure 2). Our results are in agreement with previously published data confirming that increased aortic stiffness i.e., PWV is a strong independent predictor of CV mortality in CKD/ESRD patients (8, 24). Importantly, in BEN group PWV values were lower than in non-BEN patients contributing to the lower risk for CV mortality. Chirinos et al. observed that increased arterial stiffness is an independent risk factor for heart failure in CKD patients (25). In our study heart failure was more frequently reported in non-BEN patients who had higher PWV than in BEN patients and because of that they required more ultrafiltration and more loop diuretics.

In all decade subgroups PWV values and proportion of patients with PWV above the cut off values were lower in BEN than in non-BEN group confirming our presumption that vascular aging is slower in BEN than in other ESRD patients. Less obesity, diabetes and milder disturbances in mineral metabolism contributed to lower PWV in BEN patients. However, in multivariate linear regression models adjusted to various confounders and risk factors, BEN *per se*, as a diagnosis, was the most important negative predictor of increased arterial stiffness. Early vascular aging and premature CV mortality are well-known characteristics of CKD starting from early stages (3–10, 26). Our results on lower PWV, slower increase of arterial stiffness by aging, slower progression of CKD and lower CV mortality in BEN patients compared to other ESRD patients indicate that vascular aging might be slower in BEN, and it seems that in this aspect BEN is different from other ESRD and quite contrary to adult polycystic kidney disease could be considered as a case of slower vascular aging among other CKD patients. Older age of BEN patients compared to non-BEN patients when starting with dialysis (67 vs. 57 years) could be considered as an indicator of slower vascular aging. During the study period (the same dialytic protocol was applied to both groups) patients with BEN had better control of hypertension, calcium, and phosphate which contributed to slower progression of arterial stiffness i.e., lower PWV at the end of follow-up and significantly less CV deaths in BEN group than in non-BEN group (31 vs. 12).

This is the first study which analyzed arterial stiffness and CV outcome in BEN patients. In this longitudinal study we confirmed that arterial stiffness and consecutively CV morbidity and mortality were lower in BEN patients than in other ESRD patients undergoing dialysis.

Our work has several limitations. First, in this study we have not enrolled patients in the early phases of BEN. However, as incidence of BEN is, due to improvement in agriculture practice, decreasing and disease is diminishing it would be hard to detect new BEN cases in early phase of CKD (17, 27). Furthermore, our aim was to analyze PWV in ESRD and compare BEN and non-BEN patients in this CKD stage. Second, we have enrolled BEN patients from only two BEN countries (Croatia and Bosnia and Herzegovinia). However, as the causative agent and the clinical course are the same in all countries harboring BEN it could be assumed that results from other BEN countries would be the same. Nevertheless, we are encouraging colleagues from other countries to join us in this research program. Third, one could argue why patients with aristolochic acid nephropathy from other world regions were not included. Due to large amount of toxin ingested in shorter period of time patients with iatrogenic aristolochic acid nephropathy, contrary to BEN patients, have more rapid clinical course and significantly shorter predialytic period. So, changes on their large vessels would be different from BEN, an environmental form of aristolochic acid nephropathy where low doses of aristolochic acid were ingested for decades. Nevertheless, it will be interesting to analyze arterial stiffness in this group of patients, particularly from Taiwan and China where patients sometimes were exposed to smaller amount of toxin during the longer period of time. Fourth, only office BP data were analyzed. Unfortunately, we have no data on ABPM which would additionally improve obtained results. Fifth, PWV was determined using Arteriograph, device which is validated and considered reliable (28–30). However, there are no referral cut off values for CKD patients, so we used values obtained and determined in hypertensive patients.

In this study we found slower pulse wave velocity in patients with BEN compared to other ESRD patients undergoing chronic hemodialysis and we concluded that vascular aging was slower and importantly all cause and cardiovascular mortality were lower in this group of patients comparing to other ESRD patients. Later onset of hypertension during predialytic clinical course in BEN and milder forms in advanced stages of CKD are the most plausible explanations underlying the importance of strict blood pressure control in all chronic kidney disease patients starting from early stages. In BEN lower prevalence of hypertension and milder stages could be attributed to salt-wasting as a consequence of nephrotoxic effect of aristolochic acid. Additionally, calcium, phosphate and parathyroid hormone values were better controlled in BEN patients undergoing chronic hemodialysis than in non-BEN patients what further lessened the risk of vascular calcification and progression of arterial stiffness.

BEN could be considered as a model of slower vascular aging in CKD and a lecture underscoring that good blood pressure control, adequate control of calcium and phosphate could significantly slower progression of arterial stiffness leading to lower all-cause and cardiovascular mortality in chronic kidney disease.

## Author contributions

All authors listed have made a substantial, direct and intellectual contribution to the work, and approved it for publication.

### Conflict of interest statement

The authors declare that the research was conducted in the absence of any commercial or financial relationships that could be construed as a potential conflict of interest.
